# Novel oestrogen receptor β-selective ligand reduces obesity and depressive-like behaviour in ovariectomized mice

**DOI:** 10.1038/s41598-017-04946-5

**Published:** 2017-07-05

**Authors:** Daimei Sasayama, Nobuhiro Sugiyama, Shigeru Yonekubo, Akiko Pawlak, Hiroyasu Murasawa, Mie Nakamura, Morimichi Hayashi, Takashi Ogawa, Makoto Moro, Shinsuke Washizuka, Naoji Amano, Kazuhiro Hongo, Hideki Ohnota

**Affiliations:** 1 Department of Psychiatry, Shinshu University School of Medicine, Matsumoto, Nagano 390-8621 Japan; 2 Department of Applied Occupational Therapy, Shinshu University School of Health Sciences, Matsumoto, Nagano 390-8621 Japan; 3 Discovery Research I, R&D, Kissei Pharmaceutical Co., Ltd., 4365-1 Kashiwabara, Hotaka, Azumino, Nagano 399-8304 Japan; 4 Nihon Bioresearch Inc. 6-104, Majima, Fukuju-cho, Hashima, Gifu 501-6251 Japan; 5 Safety Research Laboratory, R&D, Kissei Pharmaceutical Co., Ltd., 2320-1 Maki, Azumino, Nagano 399-8304 Japan; 6 Biologics Research, R&D, Kissei Pharmaceutical Co., Ltd., 4365-1 Kashiwabara, Hotaka, Azumino, Nagano 399-8304 Japan; 7 Department of Drug Discovery Science, Shinshu University School of Medicine, Matsumoto, Nagano 390-8621 Japan

## Abstract

Hormonal changes due to menopause can cause various health problems including weight gain and depressive symptoms. Multiple lines of evidence indicate that oestrogen receptors (ERs) play a major role in postmenopausal obesity and depression. However, little is known regarding the ER subtype-specific effects on obesity and depressive symptoms. To delineate potential effects of ERβ activation in postmenopausal women, we investigated the effects of a novel oestrogen receptor β-selective ligand (C-1) in ovariectomized mice. Uterine weight, depressive behaviour, and weight gain were examined in sham-operated control mice and ovariectomized mice administered placebo, C-1, or 17β-oestradiol (E2). Administration of C-1 or E2 reduced body weight gain and depressive-like behaviour in ovariectomized mice, as assessed by the forced swim test. In addition, administration of E2 to ovariectomized mice increased uterine weight, but administration of C-1 did not result in a significant increase in uterine weight. These results suggest that the selective activation of ERβ in ovariectomized mice may have protective effects against obesity and depressive-like behaviour without causing an increase in uterine weight. The present findings raise the possibility of the application of ERβ-ligands such as C-1 as a novel treatment for obesity and depression in postmenopausal women.

## Introduction

Postmenopausal women have a higher prevalence of obesity than premenopausal women^1^. Ovariectomized (OVX) mice exhibit weight gain that is inhibited by 17β-oestradiol (E2) treatment, suggesting that E2 depletion plays a major role in the occurrence of postmenopausal obesity^2^. Consistent with this idea, accumulating evidence shows that hormone replacement therapy (HRT) reduces abdominal obesity in postmenopausal women^3^.

The oestrogen receptors (ERs)—ERα and ERβ—function as ligand-dependent transcription factors and are activated by the binding of E2. The inhibitory effect of oestradiol on food intake and body weight in OVX rats is blocked by ERβ antisense oligodeoxynucleotides, but not by ERα antisense oligodeoxynucleotides, indicating that ERβ is associated with the anorectic action of estrogen^4^. A previous study showed that ERβ-selective ligands attenuate weight gain in OVX high-fat diet mice^5^. Together, these findings suggest that ERβ agonists may function to prevent postmenopausal obesity.

The transition to menopause is considered a period with increased risk of depression in women^6^. Recent evidence suggests that HRT with E2 may have an antidepressant effect in women with perimenopausal depression^7^. Some studies suggest the possible efficacy of raloxifene, a selective oestrogen receptor modulator (SERM), in the treatment of postmenopausal depression^8–10^. Animal studies have consistently shown that ovariectomy increases depressive-like behaviour, which can be reversed with the administration of E2^11–13^. Yang *et al*.^14^ showed that in OVX rats depressive behaviour is decreased by administration of an ERβ-selective agonist but not an ERα-selective agonist. Previously, we thoroughly examined the expression of ERα and ERβ in the mouse brain using immunohistochemical analysis^15^. We found that ERβ but not ERα is strongly expressed in the dorsal raphe (DR), which is involved in fear, anxiety, and depression. In ovariectomized (OVX) WT and in ERβ −/− mice, there was a marked reduction in the number of serotonergic neurons (tryptophan hydroxylase–positive neurons detected by immunohistochemistry)^16^. These neuronal changes in OVX mice were prevented by the selective ERβ agonist. 17β-oestradiol also prevented such neuronal changes, but the effect was smaller than that of the ERβ-specific agonist^16^. These findings suggest that ERβ may act as a potential target for the treatment of postmenopausal depression^15, 16^.

Oestrogen increases the risk of endometrial hyperplasia and endometrial cancer when prescribed without progesterone to postmenopausal women^17, 18^. However, recent data suggest a possible tumour-suppressive role of ERβ^19–23^. Moreover, some studies indicate that activation of ERβ alone does not result in increased uterine weight^24, 25^. Therefore, selective activation of ERβ may be a safer approach to HRT than the common use of oestrogen, with respect to the risk of developing endometrial hyperplasia and cancer^26^.

**Figure 1:**
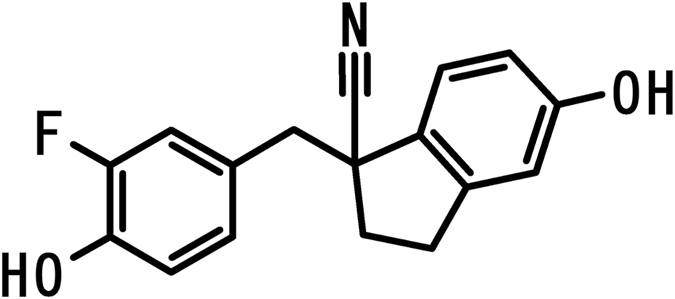
Chemical structure of C-1.

We have previously reported a novel ERβ-selective ligand, C-1 (1-(3-fluoro-4-hydroxybenzyl)-5-hydroxy-2,3-dihydro-1*H*-indene-1-carbonitrile) (Fig. 1)^27^. C-1 has a 256-fold higher agonistic selectivity for ERβ than ERα (Tables 1 and 2)^27^. To elucidate the potential effects of ERβ activation in postmenopausal women, we investigated the impact of C-1 in OVX mice. We examined uterine weight, depressive-like behaviour, and obesity in sham-operated control mice and OVX mice administered placebo, C-1, or E2.

**Table 1:** Binding affinities and transcriptional activities for ERs and ERβ selectivity of compound C-1.

RBA^a^ (%)			Transcriptional activity EC_50_^c^ (nM)		
hERα	hERβ	β/α^b^	hERα	hERβ	Rel. β/α^d^
3.5 ± 1.5	32.1 ± 5.3	9.3	1,820 ± 433	0.91 ± 0.24	256

Binding affinities for ERs were determined by a fluorescence polarization displacement assay, and are expressed as RBA values compared to the affinity of E2. Transcription activities were determined by a cell-based ERs assay. pGL4.27-(ERE)3-Luc and expression vector containing ERα or ERβ were transfected into HEK293T cells. Luciferase activity was measured and the transcriptional activity of each compound was normalized by the hypothetical maximal response of E2 (=100). ^a^RBA values were calculated by IC_50_[E2]/IC_50_[C-1] × 100 (RBA of E2 = 100), shown as mean ± SD of three independent experiments, performed in duplicate. ^b^β/α values = ERβ-RBA/ERα-RBA (ERβ selectivity of binding affinity). ^c^EC_50_ values are shown as mean ± SD of three or four independent experiments, performed in duplicate. ^d^Rel.β/α values = {ERβ-EC_50_[E2]/ERβ-EC_50_[C-1]}/{ERα-EC_50_[E2]/ERα-EC_50_[C-1]} (ERβ selectivity of agonistic activity). Detailed information can be found in ref. 27. *Abbreviations*: *EC50*, half maximal (50%) effective concentration; *hER*, human estrogen receptor; *RBA*, relative binding activity.

**Table 2:** Very low affinity of C-1 for other nuclear receptors.

Nuclear Receptor	hPR	hAR	hGR
Radioligand	[^3^H] progesterone 0.5 nM	[^3^H] methyltrienolone 1 nM	[^3^H] dexamethasone 1.5 nM
Inhibition (%) in the presence of 1 µM C-1	14.7%	−2.3%	4.4%

The binding inhibition rate (%) of compound C-1 for PR, AR, and GR was determined. Each recombinant human nuclear receptor was incubated with the indicated concentration of the radiolabeled ligand and 1 µM C-1 for 6–20 hr at 4 °C. Inhibitory effects of C-1 were determined by scintillation counting. These results show very low affinities of C-1 for other nuclear receptors. *Abbreviation*: *AR*, androgen receptor; *GR*, glucocorticoid receptor; *PR*, progesterone receptor.

**Figure 2:**
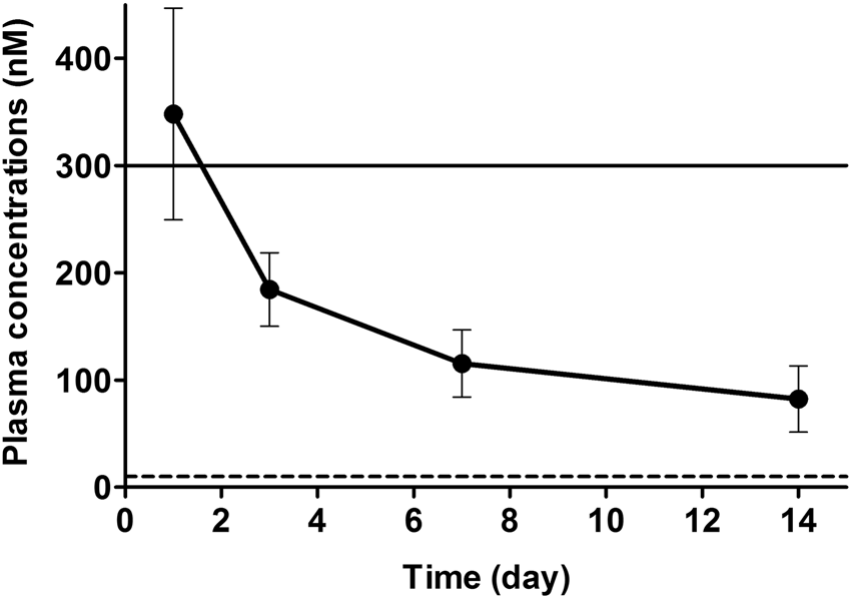
Plasma concentration of C-1 in mice after administration of sustained-release pellets of C1. Plasma concentrations of C-1 are shown as the mean with SD (n = 10). The upper solid line represents a concentration of 300 nM, at which C-1 did not show an agonistic activity for ERα *in vitro*. The lower dashed line represents a concentration of 10 nM, at which C-1 shows full agonistic activity equal to that of E2 for ERβ *in vitro*.

The *in vitro* data from our previous study^27^ showed that a serum C1 concentration of 10 nM–300 nM sufficiently activated ERβ with minimal effect on ERα. We have prepared sustained-release C1 pellets that maintain a serum concentration within this range in mice for 2 weeks (Fig. 2). These pellets were used to administer C-1 in this study.

## Results

### Uterine weight

**Figure 3:**
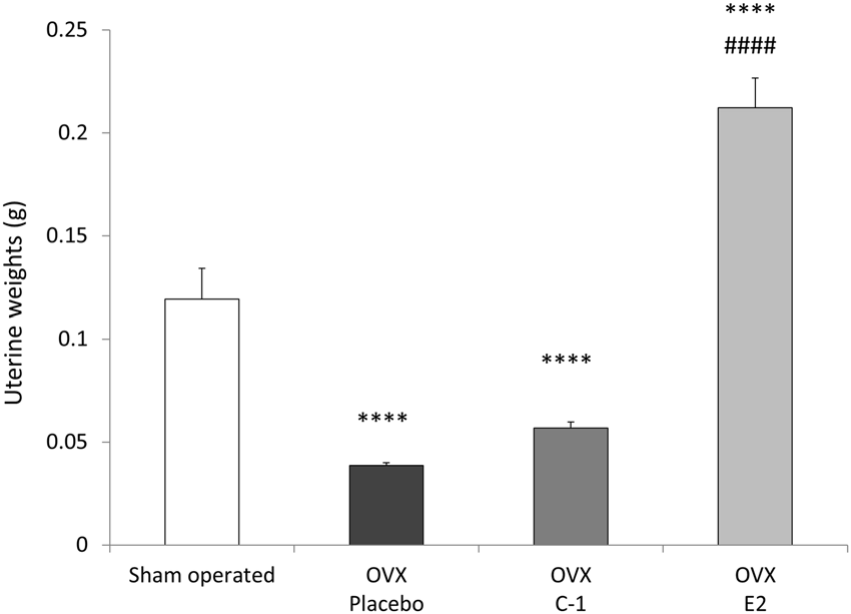
Uterine weights of sham-operated mice and ovariectomized mice administered placebo, C-1, or E2. Compared with placebo-administered mice, ovariectomized E2-administered mice but not C-1-administered mice, had higher uterine weight. Error bars represent standard errors of the mean. Post-hoc LSD test: ****P < 0.0001 as compared with sham-operated mice; ^####^ *P* < 0.0001 as compared with placebo-administered mice.

Figure 3 summarizes the uterine weights of sham-operated mice and OVX mice after 14 days of administration of placebo, C-1, or E2. A significant difference in uterine weight was observed between groups (F(3,44) = 55.50, *P* < 0.000001). Post-hoc tests showed that the uterine weights of OVX mice administered placebo and those administered C-1 were significantly lower than the uterine weights of sham-operated mice (*P* = 0.000002 and *P* = 0.00013, respectively). Conversely, the uterine weight of OVX mice administered E2 was significantly higher than that of sham-operated mice (*P* < 0.000001). No significant difference in uterine weight was found between mice administered C-1 and those administered placebo. The uterine weight was significantly greater in mice administered E2 than in those administered placebo or C-1 (*P* < 0.000001).

**Figure 4:**
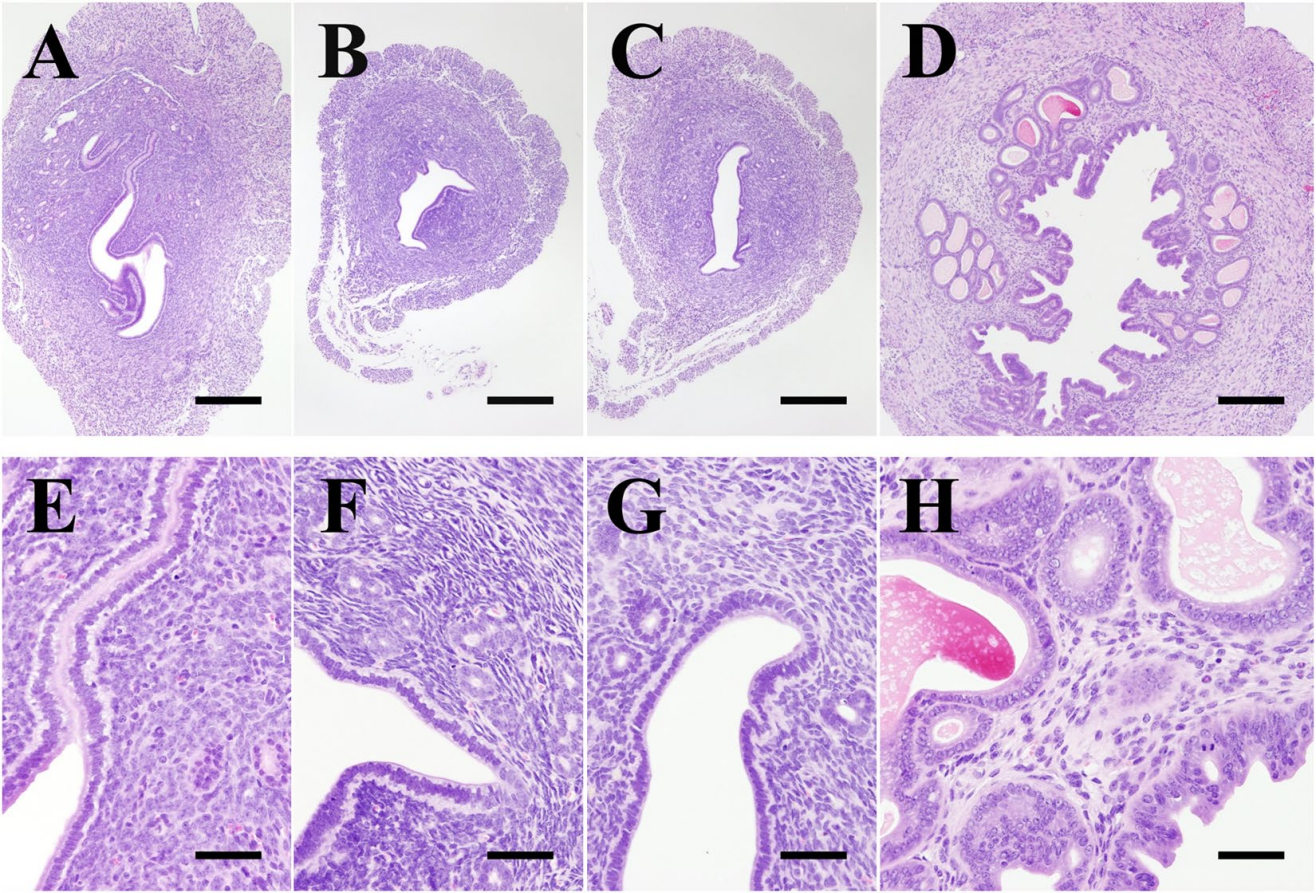
Uteri of a sham-operated mouse and OVX mice administered placebo, C-1, or E2. Uteri of a sham-operated mouse (**A**,**E**) and mice administered placebo (**B**,**F**), C-1 (**C**,**G**), or E2 (**D**,**H**) are shown. The endometrium and myometrium were atrophic in mice administered placebo or C-1. However, the endometrium and myometrium in mice administered E2 were thickened, with increased number and size of endometrial glands, and were dilated and filled with eosinophilic mucus. Scale bars = 500 µm (**A**,**B**,**C**,**D**) and 100 µm (**E**,**F**,**G**,**H**).

Figure 4 shows representative photomicrographs of H&E-stained uterine sections, which indicate that the endometrium and myometrium were thicker in mice administered E2 than in those administered C-1 or placebo. Similarly, the number and size of endometrial glands were higher in mice administered E2 than in those administered C-1 or placebo. We found no significant histological differences between the uteri of mice administered C-1 and those of mice administered placebo.

### Body weight

**Figure 5:**
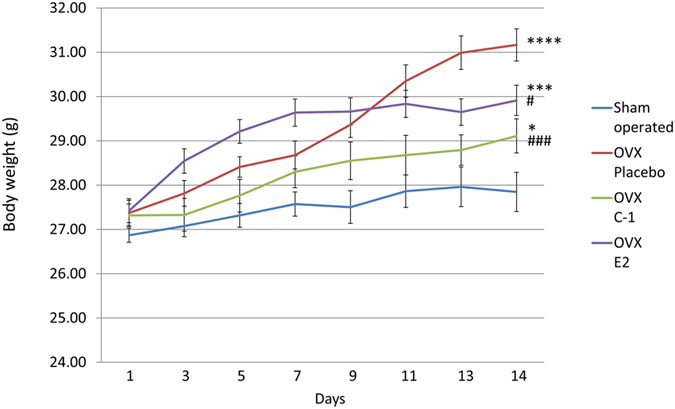
Change in body weight in sham-operated mice and ovariectomized mice administered placebo, C-1, or E2. Mice administered C-1 or E2 had significantly lower body weight at day 14 than those administered placebo. Error bars represent standard errors of the mean. Post-hoc LSD test: *P < 0.05 as compared with sham-operated mice; ***P < 0.001 as compared with sham-operated mice; ****P < 0.0001 as compared with sham-operated mice; ^#^ *P* < 0.05 as compared with placebo-administered mice; ^###^ *P* < 0.001 as compared with placebo-administered mice.

Figure 5 summarizes the body weight of each group of mice. Although no significant difference in baseline body weight (i.e., body weight at day 1) was observed between groups (F(3,44) = 1.023, *P* = 0.39), body weight at day 14 was found to be significantly different between groups (F(3,44) = 16.23, *P* < 0.000001). Post-hoc tests confirmed that body weight at day 14 was significantly greater in OVX mice than in sham-operated mice (*P* = 0.013 for mice administered C-1, *P* = 0.00012 for mice administered E2, and *P* < 0.000001 for mice administered placebo). Mice administered C-1 or E2 had significantly lower body weight at day 14 compared to mice administered placebo (*P* = 0.00013 for mice administered C-1 and *P* = 0.014 for mice administered E2). Additionally, at day 7, mice in the E2 group had significantly higher body weight than mice in the sham-operated (*P* = 0.000016), placebo (*P* = 0.029), and C-1 (*P* = 0.0030) groups. No significant difference in body weight at day 7 was observed between mice administered C-1 and mice administered placebo.

### Forced swim test

**Figure 6:**
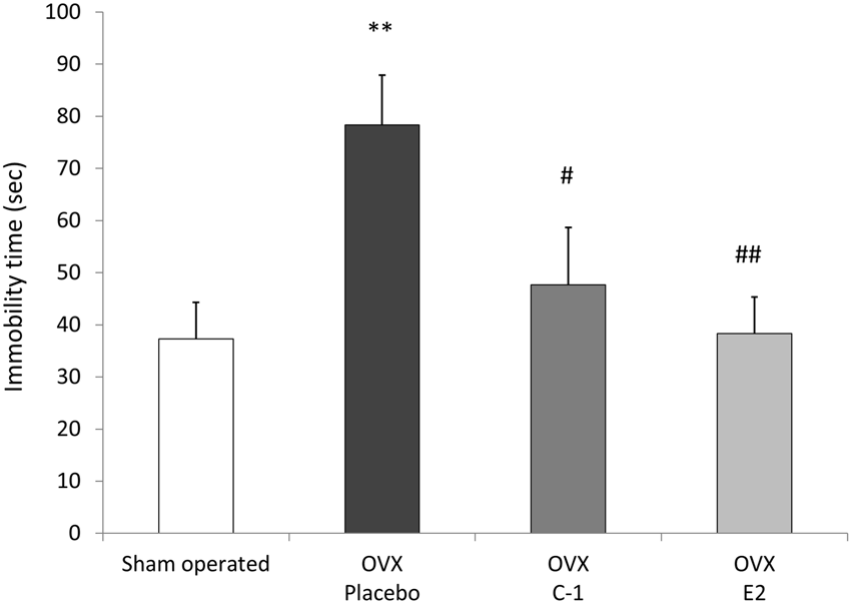
Results of forced swim test during 0–3 minutes in sham-operated mice and ovariectomized mice administered placebo, C-1, or E2. The bar graph shows the mean immobility time during 0–3 minutes of the forced swim test for control mice and ovariectomized mice administered placebo, C-1, or E2. The immobility time was significantly shorter in mice administered C-1 or E2 than in those administered placebo. Error bars represent standard errors of the mean. Post-hoc LSD test: **P < 0.01 as compared with sham-operated mice; ^#^ *P* < 0.05 as compared with placebo-administered mice; ^##^ *P* < 0.01 as compared with placebo-administered mice.

**Figure 7:**
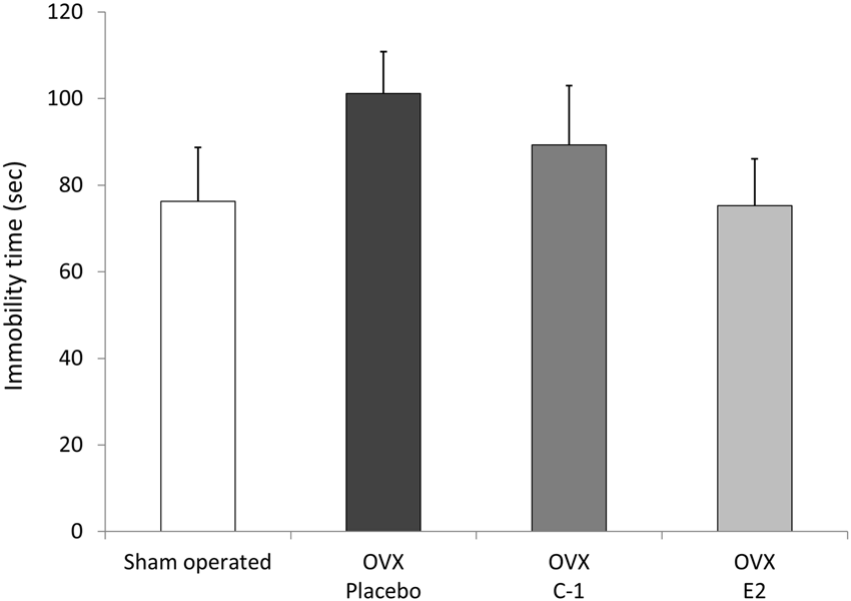
Results of forced swim test during 3–6 minutes in sham-operated mice and ovariectomized mice administered placebo, C-1, or E2. The mean immobility time during 3–6 minutes of the forced swim test is shown. No significant difference was found between groups. Error bars represent standard errors of the mean.

The immobility time during the 6 minutes of the forced swim test (FST) did not significantly differ between groups (F(3,44) = 2.66, *P* = 0.060). However, as shown in Fig. 6, a significant difference was observed in the immobility time between groups (F(3,44) = 4.76, *P* = 0.0059) during the first 3 minutes of FST. Post-hoc tests showed that the immobility time was significantly shorter in mice administered C-1 or E2 than in those administered placebo (*P* < 0.018 for mice administered C-1 and *P* < 0.0025 for mice administered E2). The immobility time of the sham-operated mice was not significantly different from that of the OVX mice administered C-1 or E2. Figure 7 shows the immobility time during the last 3 minutes of FST. No significant difference in immobility time was observed between groups (F(3,44) = 1.09, *P* = 0.36). Supplementary Table S1 provides the details of the results of the FST.

## Discussion

The present study showed that the administration of C-1 or E2 in OVX mice reduced body weight and depressive-like behaviour, as assessed by FST. However, the administration of E2 to OVX mice increased uterine weight gain as opposed to the administration of C-1. These findings suggest that the selective activation of ERβ in OVX mice may have protective effects against obesity and depression without causing an increase in uterine weight.

The most profound difference observed between E2-administered and C-1-administered mice was the change in uterine weight. Our findings corroborate the results of previous studies showing that the activation of ERα but not that of ERβ results in increased uterine weight^24, 25^. Uncontrolled uterine epithelial cell proliferation may lead to a pathological condition such as endometrial hyperplasia, which is a premalignant lesion of endometrial cancer^28^. The present finding suggests that C-1, in contrast to E2, has a small effect on uterine epithelial cell proliferation, and the administration of C-1 as opposed to that of E2 confers a lower risk of developing endometrial hyperplasia or cancer.

Our finding that both C-1 and E2 attenuate weight gain in OVX mice is consistent with results of previous studies that have used ERβ-selective ligands^5^ and E2^2^. Thus, the activation of ERs is effective in preventing weight gain in OVX mice. Several mechanisms may account for this effect. Our results showed that E2, which binds with similar affinity to ERα and ERβ, was effective in alleviating weight gain; however, the selective activation of ERβ with C-1 resulted in an even stronger effect in preventing weight gain compared to E2. The role of ERβ in the central nervous system, which is associated with anorectic action^4^, provides a plausible explanation of these observations. Additionally, mice administered E2 gained more weight than the mice administered placebo during the first 7 days of administration—a phenomenon not observed in mice administered C-1. The difference in the pattern of weight gain observed between C-1- and E2-administered mice could be due to the different effects of ERα and ERβ on appetite and energy metabolism. Therefore, to delineate the precise effects of each ER subtype, a comparison of the effects of ERα- and ERβ-selective ligand in future studies is necessary. The effects of ERs on body weight and fat distribution may differ between humans and mice; therefore, further studies are required to elucidate the effects of C-1 in humans. Nevertheless, our results have shown favourable outcomes with the administration of C-1, suggesting that the use of ERβ-selective ligands could be a potential treatment strategy for postmenopausal obesity.

The results of the FST in our study are consistent with the findings of Bekku *et al*.^29^. They showed that OVX mice exhibit prolonged immobility in FST that was prevented by the administration of E2; however, their study did not report the immobility time during the first 2 minutes of FST. The first 2 minutes of FST can be considered an acclimation period and is often excluded from analysis^30^, but many studies emphasize the significance of assessing the first few minutes of FST. Cryan *et al*.^31^ examined the effects of antidepressant treatments on the behaviour of rats during FST and showed that the most prominent effects of treatments were observed during the first 5 minutes of FST. Depressive-like behaviour induced by physical activity in rats was also observed only during the first 2 minutes of FST^32^. Another study showed that increased depression-related behaviour in TEPA-treated mice was most evident during the first 3 minutes of FST^33^. Similarly, our results showed that the immobility time during the first 3 minutes of FST was higher in OVX mice than in sham-operated mice, and administration of C-1 or E2 significantly decreased the immobility time in OVX mice. The immobility time during the last 3 minutes of FST also showed a similar pattern; however, the between-group differences did not reach statistical significance.

The present finding is in line with previous reports showing that ERβ-selective agonists produce antidepressant effects in OVX animals^14, 34^. Several mechanisms may be involved in the antidepressant effect of C-1. One is the oestrogen-related change in serotonergic activity in the brain. A previous study^35^ showed that the expression levels of genes encoding proteins supporting serotonin neurotransmission, such as 5HT1A, serotonin transporter, and tryptophan hydroxylase (TPH), were decreased after OVX in the dorsal raphe of female macaques. We have reported that ERβ−/− mice showed decreased expression of TPH in the dorsal raphe and that administration of an ERβ-selective agonist restored the expression level of TPH^16^. These findings suggest that the activation of ERβ is required for serotonergic activation in the dorsal raphe, which is the main production centre of serotonin in the brain. Therefore, administration of C-1 may exert antidepressant effects by modulating expression levels of genes associated with the serotoninergic system in the brain. Another mechanism of antidepressant action may be due to the inhibitory effect on stress-induced cortisol secretion. Patients with recent-onset depression are known to display exaggerated cortisol responses to stress^36, 37^. Weiser *et al*.^34^ showed that administration of an ERβ-selective agonist but not an ERα-selective agonist significantly lowered serum cortisol levels in OVX rats after the FST. Such modulation of the hypothalamic-pituitary-adrenal axis may be one of the mechanisms of the antidepressant effect of C-1.

In summary, a novel ERβ-selective ligand, C-1, and E2 were effective in reducing body weight gain and FST immobility time in OVX mice. However, unlike E2, C-1 did not increase uterine weight. These findings suggest that selective activation of ERβ in OVX mice has protective effects against obesity and depression without inducing a uterine proliferative response. If similar effects occur in humans, ERβ-ligands such as C-1 may become a novel choice of treatment for obesity and depression in postmenopausal women.

## Methods

### Pharmacokinetic study in mice

Female ICR mice were purchased from Charles River Laboratories Japan, Inc. (Kanagawa, Japan). Throughout the study, the animals were housed in a constant-temperature room with a 12-h/12-h lighting cycle (lights on 8:00 a.m. to 8:00 p.m.) and allowed access to laboratory chow diet (CE-2 pellets; CLEA Japan, Inc.) and water ad libitum. This study was approved by the Laboratory Animal Committee of Kissei Pharmaceutical Co., Ltd. The experiments were conducted in accordance with the Guidelines for Management and Welfare of Experimental Animals (the Laboratory Animal Committee of Kissei Pharmaceutical Co., Ltd.).

Pharmacokinetic studies were performed in female ICR mice (7-week-old) after subcutaneous insertion of C-1 containing pellets. Blood samples were collected at 1, 3, 7, and 14 days after subcutaneous administration. The blood samples were allowed to clot at 4 °C overnight, then serum samples were prepared by centrifugation at 2,280 × *g* for 10 min at 4 °C. The collected serum samples were stored at −20 °C until analysis. Samples were analysed using a LC system coupled to an API4000 mass spectrometer (SCIEX, Toronto, Canada). For MS detection, the Turbo V ion source was operated in negative ion mode. LC separation was performed using a NexeraX2 system (Shimadzu, Kyoto, Japan) with a gradient elution from a Cadenza CD-C18 HT column (3 μm, 50 mm × 2.0 mm ID; Imtakt, Kyoto, Japan). The flow rate and column temperature were set at 0.5 ml/min and 50 °C, respectively. The mobile phase consisted of solvent A, 10 mM ammonium acetate aqueous solution and solvent B, acetonitrile containing 0.1% formic acid.

### Animals for forced swim test

Female Jcl:ICR mice at 7 weeks of age were obtained from CLEA Japan, Inc. (Japan). Mice were maintained in a temperature-controlled room at 23 ± 3 °C and humidity of 55 ± 15% with a 12-h light/12-h dark cycle (lights on at 6.00 a.m.) and fresh air changes every 5 minutes. Mice were acclimatized to laboratory conditions for at least 14 days prior to experiments and had free access to pellet diet (CRF-1; Oriental Yeast Co., Japan) and water. Five days before ovariectomy, the diet was changed to modified AIN93G pellets, which contain corn oil instead of soy oil, specially prepared for this experiment (Oriental Yeast, Japan). This study was approved by the Animal Care and Use Committee of Nihon Bioresearch Inc., accredited by the Center for Accreditation of Laboratory Animal Care and Use of Japan Health Sciences Foundation. The experiments were conducted in accordance with the Guidelines for Management and Welfare of Experimental Animals (Hashima Laboratory, Nihon Bioresearch Inc., April 2, 2007; modified on August 27, 2010).

### Ovariectomy protocol

The ovariectomy was performed bilaterally under isoflurane anaesthesia in 36 randomly assigned mice at 9 weeks of age. Briefly, the ovarian fat pad was lifted through the dorsal incision and the ovary was exteriorized and carefully removed. Sham-operated animals underwent the same procedure as the ovariectomized mice but without resection of the ovaries. These surgeries were performed at Nihon Bioresearch Inc. (Gifu, Japan).

### Treatment protocol

Ovariectomized mice were randomly assigned to one of the following three groups (n = 12 per group): (1) mice implanted with placebo pellets, (2) mice implanted with C-1 pellets (15.87 mg·kg^−1^·d^−1^, 10 mg/pellet/21-day release), and (3) mice implanted with E2 pellets (10.5 µg·kg^−1^·d^−1^, 6.615 µg/pellet/21-day release). C-1 was synthesized as described previously^27^. C1 pellets were developed to maintain a plasma concentration within the range (i.e. 10 nM–300 nM) at which C1 activates ERβ with a minimal effect on ERα (Fig. 2) for two weeks. A similar dose (6 µg·kg^−1^·d^−1^) of E2 was proven to have long-term effects on body and uterine weights and bone mineral density when dosed for 6 weeks^38^. The same E2 pellets were used in our previous study^16^. The duration of administration was 2 weeks because similar studies^29^ were conducted in 2 weeks. All pellets, including placebo, were purchased from Innovative Research of America, Sarasota, FL, USA. The pellets were implanted subcutaneously in the back of the neck under isoflurane anaesthesia.

### Body weight measurement

The animals were weighed every other day and on the day of FST. One of the following precision balances was used: PB3002, PG2002-S, PB3002-S/FACT, or MS1602S/02 (Mettler Toledo, Tokyo, Japan).

### Forced swim test (FST)

FST was performed as previously described^39^. Briefly, on day 14 of administration, the mice were placed in tanks filled with water (diameter: 145 mm, height: 190 mm, depth of water: 100 mm, water temperature: approximately 24 ± 2 °C). Swimming time was measured using an itching measurement system (Neuroscience Inc., Japan) by attaching magnets to the hind legs of mice and measuring the movement of hind legs. Swimming time was analysed every minute.

### Collection of uterus

After FST, mice were sacrificed under isoflurane anaesthesia and uteri were removed and weighed using precision balances. Uteri were fixed in 10% neutral buffered formalin and processed for 3 µm paraffin sections. Sections were dewaxed in Lemosol A (Wako Pure Chemical Industries, Osaka, Japan) and then rehydrated in graded concentrations of ethanol, followed by H&E staining for histological examination.

### Statistical analysis

The data were analysed using one-way analysis of covariance (ANOVA). The least significant difference (LSD) test was used as a post-hoc test for paired comparisons. *P* < 0.05 was considered significant.

## Electronic supplementary material

Supplementary Table S1
