# Supplementary material for: Novel oestrogen receptor β-selective ligand reduces obesity and depressive-like behaviour in ovariectomized mice
Source: Sci Rep. 2017 Jul 5;7:4663. doi: 10.1038/s41598-017-04946-5 (PMC5498485; doi:10.1038/s41598-017-04946-5)
Supplement: Supplementary file 1 — Supplementary Table S1 [file 41598_2017_4946_MOESM1_ESM.pdf]

Supplementary information

Novel oestrogen receptor β-selective ligand reduces obesity and depressive-like behaviour in ovariectomized mice

Daimei Sasayama[1], Nobuhiro Sugiyama[1, 2 *], Shigeru Yonekubo[3], Akiko Pawlak[4], Hiroyasu Murasawa[4], Mie Nakamura[4], Morimichi Hayashi[5], Takashi Ogawa[5], Makoto Moro[6], Shinsuke Washizuka[1], Naoji Amano[7], Kazuhiro Hongo[7], Hideki Ohnota[7]

[1] Department of Psychiatry, Shinshu University School of Medicine, Matsumoto, Nagano, 390-8621, JAPAN

[2] Department of Applied Occupational Therapy, Shinshu University School of Health Sciences, Matsumoto, Nagano, 390-8621, JAPAN

[3] Discovery Research I, R&D, Kissei Pharmaceutical Co., Ltd., 4365-1 Kashiwabara, Hotaka, Azumino, Nagano, 399-8304, JAPAN

[4] Nihon Bioresearch Inc. 6-104, Majima, Fukuju-cho, Hashima, Gifu, 501-6251, JAPAN

[5] Safety Research Laboratory, R&D, Kissei Pharmaceutical Co., Ltd., 2320-1 Maki, Azumino, Nagano, 399-8304, JAPAN

[6] Biologics Research, R&D, Kissei Pharmaceutical Co., Ltd., 4365-1 Kashiwabara, Hotaka, Azumino, Nagano, 399-8304, JAPAN

[7] Department of Drug Discovery Science, Shinshu University School of Medicine, Matsumoto, Nagano, 390-8621, JAPAN

Corresponding author and person to whom reprint requests should be addressed:

Professor Nobuhiro Sugiyama, MD, PhD

Supplementary Table S1: Immobility time in the forced swim test.

Group	Animal	Immobility time (s)								
		0-1 min	1-2 min	2-3 min	3-4 min	4-5 min	5-6 min	Total (0-6 min)	Total (0-3 min)	Total (3-6 min)
Sham-operated mice	Sham-op mouse 1	0.63	14.22	42.33	31.44	37.87	48.82	175.31	57.18	118.13
	Sham-op mouse 2	2.24	8.75	6.49	15.05	16.41	9.31	58.25	17.48	40.77
	Sham-op mouse 3	0.00	41.83	17.45	17.62	20.48	38.22	135.60	59.28	76.32
	Sham-op mouse 4	2.99	3.30	7.87	42.96	26.09	30.57	113.78	14.16	99.62
	Sham-op mouse 5	5.14	11.08	20.68	26.88	17.58	22.14	103.50	36.90	66.60
	Sham-op mouse 6	0.00	9.71	14.63	12.37	26.51	27.41	90.63	24.34	66.29
	Sham-op mouse 7	0.01	1.32	2.29	2.82	0.00	8.98	15.42	3.62	11.80
	Sham-op mouse 8	2.37	3.32	19.26	22.97	21.66	20.61	90.19	24.95	65.24
	Sham-op mouse 9	1.97	6.19	31.28	20.49	19.19	18.96	98.08	39.44	58.64
	Sham-op mouse 10	0.00	23.27	25.41	45.42	34.68	36.50	165.28	48.68	116.60
	Sham-op mouse 11	7.88	11.29	9.59	9.51	7.84	11.45	57.56	28.76	28.80
	Sham-op mouse 12	10.17	30.18	52.28	58.96	58.93	48.69	259.21	92.63	166.58
OVX placebo-administered mice	OVX mouse 1	10.60	24.74	31.40	26.70	33.95	40.19	167.58	66.74	100.84
	OVX mouse 2	5.30	20.61	31.64	39.18	39.37	28.04	164.14	57.55	106.59
	OVX mouse 3	44.50	37.74	34.42	36.83	49.41	33.92	236.82	116.66	120.16
	OVX mouse 4	43.64	43.85	52.10	50.04	39.02	40.24	268.89	139.59	129.30
	OVX mouse 5	7.07	29.76	34.59	1.40	28.54	32.18	133.54	71.42	62.12
	OVX mouse 6	12.58	26.36	45.89	37.11	20.84	35.72	178.50	84.83	93.67
	OVX mouse 7	30.61	22.70	30.96	24.33	35.97	38.69	183.26	84.27	98.99
	OVX mouse 8	23.67	19.10	14.40	21.58	11.87	25.13	115.75	57.17	58.58
	OVX mouse 9	19.16	8.94	15.44	5.78	18.37	22.48	90.17	43.54	46.63
	OVX mouse 10	0.00	1.85	20.34	20.76	27.00	50.15	120.10	22.19	97.91
	OVX mouse 11	22.07	24.17	35.47	47.81	49.94	51.15	230.61	81.71	148.90
	OVX mouse 12	23.12	41.60	49.79	51.80	48.12	51.02	265.45	114.51	150.94
OVX C-1-administered mice	OVX+C-1 mouse 1	2.44	4.76	2.23	2.46	2.67	15.43	29.99	9.43	20.56
	OVX+C-1 mouse 2	4.32	5.40	1.69	7.31	5.15	7.99	31.86	11.41	20.45
	OVX+C-1 mouse 3	5.98	7.18	10.80	22.01	16.14	33.50	95.61	23.96	71.65
	OVX+C-1 mouse 4	5.30	14.16	25.90	32.60	28.71	35.53	142.20	45.36	96.84
	OVX+C-1 mouse 5	12.62	16.82	22.78	19.22	34.28	28.90	134.62	52.22	82.40
	OVX+C-1 mouse 6	2.20	2.45	18.33	14.18	3.28	17.04	57.48	22.98	34.50
	OVX+C-1 mouse 7	4.93	41.71	44.75	53.90	49.83	54.44	249.56	91.39	158.17
	OVX+C-1 mouse 8	2.92	4.95	7.51	28.46	27.51	40.38	111.73	15.38	96.35
	OVX+C-1 mouse 9	16.64	46.23	49.83	51.56	58.62	46.43	269.31	112.70	156.61
	OVX+C-1 mouse 10	0.88	5.89	10.99	7.29	33.45	49.11	107.61	17.76	89.85
	OVX+C-1 mouse 11	11.55	41.15	55.51	50.19	59.48	23.98	241.86	108.21	133.65
	OVX+C-1 mouse 12	3.58	27.99	29.87	26.48	45.49	38.94	172.35	61.44	110.91
OVX E2-administered mice	OVX+E2 mouse 1	0.00	4.63	10.14	8.17	14.85	10.97	48.76	14.77	33.99
	OVX+E2 mouse 2	3.09	4.37	2.26	30.91	19.64	20.38	80.65	9.72	70.93
	OVX+E2 mouse 3	8.01	16.51	19.87	20.78	18.45	22.92	106.54	44.39	62.15
	OVX+E2 mouse 4	5.45	27.08	31.35	29.09	25.13	28.48	146.58	63.88	82.70
	OVX+E2 mouse 5	0.51	5.33	8.70	11.44	20.52	20.36	66.86	14.54	52.32
	OVX+E2 mouse 6	3.57	21.29	23.28	49.90	43.58	27.98	169.60	48.14	121.46
	OVX+E2 mouse 7	3.93	12.57	26.99	49.60	47.92	45.17	186.18	43.49	142.69
	OVX+E2 mouse 8	3.41	3.58	5.22	13.30	8.59	11.93	46.03	12.21	33.82
	OVX+E2 mouse 9	1.59	24.38	26.98	16.50	17.65	8.21	95.31	52.95	42.36
	OVX+E2 mouse 10	12.68	17.12	16.13	19.96	39.21	49.56	154.66	45.93	108.73
	OVX+E2 mouse 11	1.25	12.47	8.53	12.62	15.95	13.51	64.33	22.25	42.08
	OVX+E2 mouse 12	16.06	37.28	34.68	30.39	26.04	53.56	198.01	88.02	109.99
